# Effect of the human papillomavirus vaccine on the risk of genital warts: a nationwide cohort study of Korean adolescent girls

**DOI:** 10.4178/epih.e2024040

**Published:** 2024-03-18

**Authors:** Jaeyoung Cho, Eun Mi Kim, Jihye Kim, Ju-Young Shin, Eui Hyeok Kim, Jong Heon Park, Seunghyun Lewis Kwon, Geun-Yong Kwon, Soon-Ae Shin, Jaiyong Kim

**Affiliations:** 1 Department of Big Data Strategy, National Health Insurance Services, Wonju, Korea; 2 Sungkyunkwan University School of Pharmacy, Suwon, Korea; 3 Department of Obstetrics & Gynecology, CHA Ilsan Medical Center, Goyang, Korea; 4 Division of Immunization, Bureau of Healthcare Safety and Immunization, Korea Disease Control and Prevention Agency, Cheongju, Korea; 5 Division of Immunization Planning, Bureau of Healthcare Safety and Immunization, Korea Disease Control and Prevention Agency, Cheongju, Korea; 6 Health Insurance Research Institute, National Health Insurance Services, Wonju, Korea

**Keywords:** Human papillomavirus infection, Vaccines, Genital warts, Condylomata acuminata

## Abstract

**OBJECTIVES:**

The purpose of this study was to assess the effectiveness of human papillomavirus (HPV) vaccination administered to adolescent girls through Korea’s National Immunization Program.

**METHODS:**

This retrospective cohort study included patients who were 12-13 years old, whether vaccinated or unvaccinated, between July 2016 and December 2017. The incidence of genital warts (GWs) was monitored through 2021. Time-stratified hazard ratios (HRs) were estimated, adjusting for birth year, socioeconomic status, and the level of urbanization of the region, and were presented with 95% confidence intervals (CIs). Data were sourced from the Immunization Registry Integration System, linked with the National Health Information Database.

**RESULTS:**

The study included 332,062 adolescent girls, with an average follow-up period of approximately 4.6 years. Except for the first year, the HRs for the vaccinated group were lower than those for the unvaccinated group. The HRs for specific cut-off years were as follows: year 2, 0.62 (95% CI, 0.31 to 1.13); year 3, 0.58 (95% CI, 0.35 to 0.96); and year 4 and beyond, 0.39 (95% CI, 0.28 to 0.52).

**CONCLUSIONS:**

Our findings indicate that HPV vaccination was associated with a reduction in the risk of GWs among adolescent girls. Notably, this reduction became significant as the incidence of GWs increased with age.

## GRAPHICAL ABSTRACT

[Fig f3-epih-46-e2024040]

## Key Message

In this retrospective cohort study, our results demonstrated that HPV vaccination is associated with a reduction in the risk of GWs among adolescent girls. In the short term, the NIP of Korea can be considered effective in providing protection against GWs. Future studies need to analyze the impact of vaccines on more serious diseases such as precancerous lesions or cancer.

## INTRODUCTION

Vaccination against human papillomavirus (HPV) is recognized as the primary method for preventing cervical cancer globally. In 2020, the World Health Organization highlighted the importance of administering the HPV vaccine to girls aged 9-14 years as a critical measure to prevent and control cervical cancer [1]. In Korea, the HPV vaccine was incorporated into the National Immunization Program (NIP) in June 2016, with free vaccination offered to 12-year-old girls. By 2022, the NIP’s HPV vaccination program had expanded to cover all adolescent girls aged 13 years to 17 years, as well as low-income female aged 18 years to 26 years.

Genital warts (GWs) are benign growths on the anogenital skin and mucosa resulting from the sexual transmission of HPV. HPV types 6 and 11 are primarily responsible for the development of GWs, also known as condylomata acuminata. Since GWs typically develop within an average of 3 months following infection, they serve as an early clinical indicator to assess the effectiveness of the quadrivalent vaccine [2]. The highest prevalence of GWs is observed in female aged 20 years to 24 years and in male aged 25 years to 29 years. Furthermore, GWs are associated with immunosuppression, HIV infection, and a history of sexually transmitted infections [3-5].

GWs are typically diagnosed through visual identification, with colposcopy or biopsy performed as necessary. Available treatments for GWs can reduce the symptoms but do not permanently eradicate the HPV infection. Additionally, it remains unclear whether treating GWs diminishes the risk of transmitting HPV. GWs spontaneously resolve without treatment in approximately one-third of infected individuals within the first year, allowing for the option of a “wait-and-see” approach. Even when treated, GWs may recur. In fact, the recurrence rate of GWs within 3 months after treatment completion ranges from 25% to 67% [4,6,7].

The effectiveness of HPV vaccination has been demonstrated in studies estimating the prevalence rates of cervical precancerous lesions, GWs, or HPV infection. These studies indicate that HPV vaccination is effective in reducing the incidence of GWs among girls and female in their twenties [8-12]. One meta-analysis presented compelling evidence supporting the effectiveness of HPV vaccination. Specifically, the administration of the quadrivalent HPV vaccine led to a significant decrease in the prevalence of GWs among girls aged 15 years to 19 years. However, a significant reduction was not observed in 30-year-old female. The effectiveness of the vaccine appears to diminish with age, likely because a higher proportion of individuals have already been infected with HPV by the time of vaccination. Accordingly, the vaccine is most effective when administered prior to infection. High vaccination coverage rates contribute to greater population-level benefits and the herd immunity effect [13].

Currently, no national-level findings based on individual data are available regarding the effectiveness of the HPV vaccine in Korea. Therefore, this study was conducted to evaluate the effectiveness of HPV vaccination in preventing GWs among adolescent girls in Korea and to provide scientific evidence to inform a national immunization policy, drawing on individual-level data.

## MATERIALS AND METHODS

### Data

Using de-identified personal codes, a dataset was generated by linking individual records from the Immunization Registry Integration System (IRIS) with those from the National Health Information Database (NHID) for the period between July 2011 and December 2021.

To ascertain vaccination status, we utilized immunization records from July 2016 to June 2021 that were registered with the IRIS of the Korea Disease Control and Prevention Agency. These records included the date of vaccination and the type of vaccine administered. As part of the NIP, data were entered into the computerized IRIS system following vaccinations at public health centers, clinics, and hospitals. The pertinent legislation requires that all vaccination information be recorded in this system.

The NHID of the National Health Insurance Service contains claims-based data encompassing details regarding the eligibility and medical treatments of insured individuals. Eligibility was determined using extracted data such as age, sex, birth year, region, type of insurance, and income-based premium level. Underlying medical conditions were discovered using various parameters, including diagnosis, date of diagnosis, prescriptions, treatments received, and examinations conducted. International Classification of Diseases, 10th edition (ICD-10) diagnostic codes were used to identify conditions. The claims data demonstrated a positive predictive value of 82% for the accuracy of diagnostic codes within the overall inpatient data [14].

### Study design

**Figure 1.**
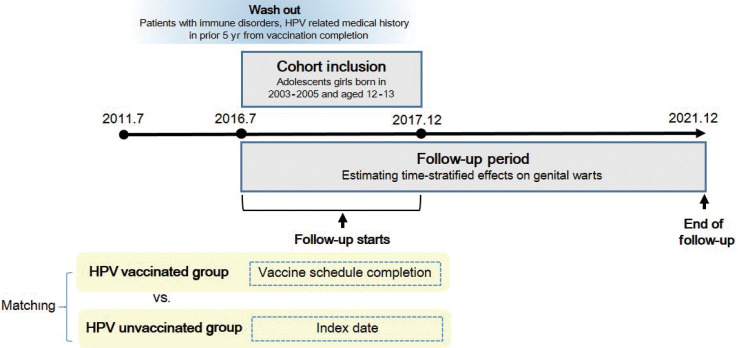
Overview of the study design. HPV, human papillomavirus.

This investigation was a retrospective cohort study that employed survival analysis methods (Figure 1). We examined the time-stratified impact of HPV vaccination on the incidence of GWs in adolescent girls. The study participants were divided into 2 groups based on their HPV vaccination status between July 2016 and December 2017: those who had completed the vaccination series and those who had not received any dose of HPV vaccination. To ensure group homogeneity, participants underwent 1:1 random matching, using birth year and socioeconomic status as exact matching variables. For the vaccinated group, the follow-up period began on the date they completed the vaccination schedule. This same date was assigned as the index date representing the start of the follow-up period for the unvaccinated group.

Follow-up was conducted using data through December 2021, with the primary outcome being GW disease. Results were stratified temporally into year 1, year 2, year 3, and year 4 and subsequent years. The washout and exclusion criteria are detailed in the Study population section.

### Study population

The research participants were adolescent girls aged 12 years to 13 years, born between 2003 and 2005, who were enrolled in the National Health Insurance program from July 2016 to December 2017. These individuals were part of the initial NIP cohort for HPV vaccination and were among the first to receive the vaccine free of charge.

The exclusion criteria for this study were as follows: (1) girls who were not of the age recommended for vaccination by the NIP during the inclusion period or who had received only partial vaccination; (2) those who had received bivalent vaccination, with GWs not included in the indication; (3) those missing essential data, including socioeconomic status (as indicated by health insurance premium level), regional characteristics (namely, the urbanization level), and date of birth; and (4) patients with immune disorders, including individuals who had undergone hematopoietic stem cell transplantation or solid organ transplantation; patients who had leukemia, multiple myeloma, or autoimmune disease; and inpatients prescribed immunosuppressants; (5) anyone with a diagnosis of HPV-related disease (Supplementary Material 1) within the 5 years prior to the start of the follow-up period or a history of undergoing tests indicative of sexual activity, such as Pap smears, sexually transmitted disease tests, and HPV tests. These exclusion criteria were established based on clinical conditions and the recommendations of clinical experts.

### Definitions of vaccinated and unvaccinated groups

The vaccinated group consisted of individuals who completed their HPV vaccination regimen between July 2016 and December 2017. Completion of the vaccination schedule was assessed using drug usage records provided by the Ministry of Food and Drug Administration, considering the age of the recipient and the type of vaccine administered. For the quadrivalent vaccine, the completion date was defined as the date of the second dose for individuals younger than 13 years and the date of the third dose for those aged 14 years or older. For the non-avalent vaccine, completion was marked by the second dose for those younger than 14 years and by the third dose for individuals aged 15 years or older. The age on the date of the initial dose was used as the reference point. For those who received vaccines of 2 or more types, the type with the earliest completion date was used. The unvaccinated group included those who remained unvaccinated for HPV between July 2016 and December 2017.

### Definition of the outcome measure

The outcome measure was the onset of GWs. This was identified by treatments under the ICD-10 code A630, as advised by clinical experts. The date of GW onset was defined as the earliest treatment date recorded within the follow-up period. Clinical practice standards dictate that GWs are typically diagnosed through visual identification, and in most cases, observation without treatment is acceptable [4,6]. Consequently, no additional criteria— such as prescriptions or treatment procedures—were applied for this study. This approach aligns with previous claims-based studies that have used the incidence of GWs as the primary outcome [8,15-17]. All information concerning the disease, including principal, secondary, and other recorded diagnoses, was reviewed.

### Control of potential confounders

We incorporated the available variables of birth year, socioeconomic status, and the urbanization level of the region as confounders associated with vaccination status, which represent risk factors for the outcome. The hazard ratio (HR) for the vaccinated group was computed by including these variables in the statistical analysis models after adjusting for their effects.

The birth years recorded included 2003, 2004, and 2005. Socioeconomic status was categorized by Medical Aid receipt along with 5 levels of income-based health insurance premiums: lowest, low, middle, high, and highest. The regional urbanization level was classified as metropolitan, urban, or rural. These variables were applied in a fixed baseline state.

### Statistical analysis

Descriptive analyses of baseline characteristics and the frequency of GWs were performed to compare the vaccinated and unvaccinated participants. Kaplan–Meier (KM) survival analysis was utilized to assess the significance of differences in the incidence of GWs between these groups.

To estimate the effect of vaccination status while adjusting for confounding factors, a Cox proportional hazards regression analysis was employed. Adjusted HRs and 95% confidence intervals (CIs) were calculated, with an alpha level of 0.05 indicating statistical significance. The hazard for the unvaccinated group was set as the reference at 1.00; therefore, an HR of less than 1.00 for the vaccinated group suggested that vaccination was effective.

The proportionality of covariates, a key assumption of the Cox proportional hazards model, was assessed using log-log plots and tests for proportionality. In this study, which employed a fixed risk factor, time dependence was a possibility. A time-dependent effect can arise when the stability of risk factors remains constant, while their effects potentially vary over time. To address this, we adopted the time-stratified HR approach described by Dekker et al. [18]. We stratified the follow-up period into intervals (years 1, 2, 3, or 4 and beyond) and calculated HRs independently for each interval. For each time interval, we defined the analyzed population by excluding girls who were diagnosed with GWs or had been censored before the start of each stratified period, along with their matched counterparts. This method ensured compliance with the Cox assumption, enabling interpretation of the estimates with the understanding that the population definition was conditional—with the retained population referred to operationally as “survivors”— for each stratified period. Data analysis was performed using SAS Enterprise Guide version 8.1 (SAS Institute Inc., Cary, NC, USA).

### Ethics statement

The study received approval from the Institutional Review Board of the National Health Insurance Service (No. 2022-HR-03-035) and was conducted in accordance with the Declaration of Helsinki and Good Clinical Practice Guidelines.

## RESULTS

### Baseline characteristics of the study cohort

**Figure 2.**
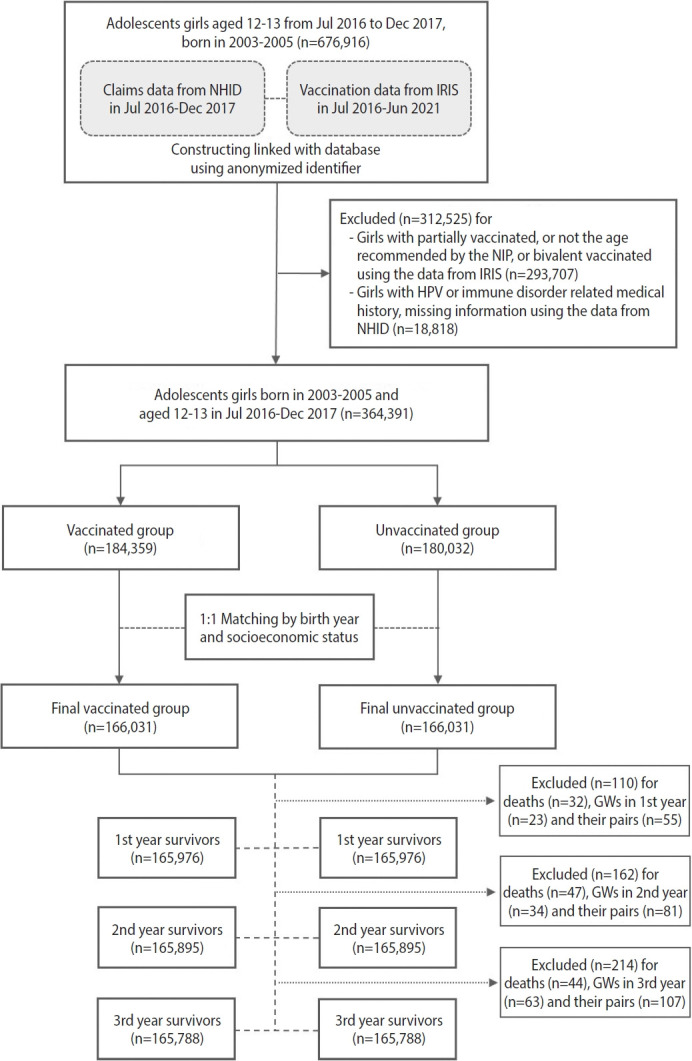
Flowchart of the enrollment procedure using a database created by linking Immunization Registry Information System (IRIS) and National Health Information Database (NHID) data. NIP, National Immunization Program; GWs, genital warts.

The study included adolescent girls born between 2003 and 2005 (n= 332,062), who were assigned in equal numbers to the vaccinated and unvaccinated groups (n= 166,031 each). The mean follow-up duration was 4.6 years for both groups. Among those in the vaccinated group, 99.9% had received the quadrivalent vaccine (Table 1 and Figure 2).

The mean age at the beginning of the follow-up period was 13.3 years, while that at the end of the period was 17.9 years. Cohorts were assigned by birth year, with 50.7% of participants born in 2003, 36.6% in 2004, and 12.7% in 2005. Regarding socioeconomic status, 22.2% belonged to the “lowest” health insurance contribution category, while 22.0% were classified in the “high” category. After matching, no significant differences were found in age, birth year, or socioeconomic status between the vaccinated and unvaccinated groups (Table 1).

### Survival analysis

During the overall follow-up period across all cohorts, KM survival analysis revealed significant differences between the survival functions of the vaccinated and unvaccinated groups, except for the cohort born in 2005. Initially, the 2 KM curves overlapped, but as follow-up continued, they began to diverge (Supplementary Material 2).

In the Cox proportional hazards regression analysis, the HR for the vaccinated participants compared to the unvaccinated group was estimated, considering adjustments for birth year, socioeconomic status, and regional urbanization level. The time-stratified HRs, calculated after confirming that the assumptions of the Cox proportional hazards model were met, are presented in Table 2.

For the first year of follow-up, the hazard was approximately 1.3 times higher for vaccinated girls compared to their unvaccinated counterparts. Through the second year of follow-up among the 1-year survivors, the hazard was roughly 38% lower for vaccinated individuals than for those who were unvaccinated; however, neither difference was statistically significant. Including the third year of follow-up for the 2-year survivors, the hazard was approximately 42% lower among vaccinated compared to unvaccinated girls (HR, 0.58; 95% CI, 0.35 to 0.96). When the analysis was extended to include the fourth year and beyond for 3-year survivors, the hazard was approximately 61% lower among vaccinated participants compared to unvaccinated girls (HR, 0.39; 95% CI, 0.28 to 0.52).

For individuals born in 2003, a significant difference in HRs was observed upon incorporating the third year of follow-up for those categorized as 2-year survivors. In this group, the hazard was approximately 52% lower for vaccinated participants compared to unvaccinated girls (HR, 0.48; 95% CI, 0.24 to 0.96). When year 4 and beyond was included for the analysis of 3-year survivors, the hazard was approximately 62% lower among vaccinated girls relative to their unvaccinated counterparts (HR, 0.38; 95% CI, 0.26 to 0.55).

For individuals born in 2004, the hazard for 3-year survivors was 56% lower among those vaccinated compared to their unvaccinated counterparts—a significant finding—when including the third year of follow-up (HR, 0.44; 95% CI, 0.26 to 0.75). Data for years 1 and 2 for the 2004 cohort and all data for the 2005 cohort are not presented, as statistical analysis was not completed due to an insufficient number of events.

## DISCUSSION

In this study, data from the IRIS were linked with information from the NHID to construct a nationwide set of individual data. Cox proportional hazards regression analysis was performed to assess the effect of HPV vaccination on the incidence of GWs in adolescent girls. The mean incubation period for GWs is 3 months, which was used as the endpoint for early monitoring to evaluate the impact of the HPV vaccination program on the population [19]. The study cohort excluded sexually active individuals based on records of an HPV-related diagnosis or records of undergoing tests indicative of sexual activity; therefore, the findings of this study reflect outcomes in a population that was HPV-naive at the time of vaccination. The effectiveness observed in this study is primarily attributable to the quadrivalent vaccine, as it was administered to nearly all vaccinated participants.

The results of the analysis indicated that the HPV vaccine was effective, except for outcomes within 1 year of follow-up. In addition to the HR for year 1 suggesting a lack of effectiveness, the HRs for years 1 and 2 (unlike those for years 3 and 4) were not significant. This suggests that the vaccine’s effectiveness becomes more pronounced after the early follow-up period. The absence of a significant difference through year 2 may be attributed to the relatively low incidence of GWs for comparison, stemming from the participants’ younger age. However, given that significant and consistent differences emerged after this point, we anticipate clearer results in the future. The findings of this study align with previous research, which showed significant reductions in the prevalence of GWs among both adolescent girls and female in their early twenties following HPV vaccination [20,21]. A prior meta-analysis found that vaccinated girls aged 15 years to 19 years had a lower risk of developing GWs compared to their unvaccinated peers, and the vaccine was more effective in this age group than among female in their twenties. This disparity is likely because the younger group was more likely to be vaccinated before being exposed to the virus [21]. What distinguishes this study from earlier ones is the use of individual data rather than pooled prevalence data. Additionally, the effect size estimated in our study, which utilized real-world data, was smaller than that observed in a previous randomized control trial (RCT) [19]. With RCTs, efficacy is assessed in a strictly controlled environment, while real-world evidence is more valuable for post-marketing surveillance. This can result in differences in effect size.

Research examining adolescent girls in Australia and Germany also indicated a reduction in the incidence of GWs 1 year after the introduction of national HPV vaccination programs [12,22]. In research involving female participants aged 14 years to 23 years, the mean time to the onset of GWs was 5.3 years after vaccination, which aligns with the findings of this study [17]. An RCT using Cox regression analysis also reported time-dependent results [19]. Furthermore, a systematic review noted a decline in the rate of GW diagnosis among girls and female aged 15-24 years within 2 years after receiving the quadrivalent HPV vaccine [21].

The time-dependent nature of the results may relate to the HPV exposure status of the study participants. The adolescent girls in this study belonged to an age group with a low likelihood of engaging in sexual activity within the first 2 years of follow-up. As the follow-up duration increased, so did the participants’ age, thereby raising the risk of HPV exposure due to the initiation of sexual activity. This increase in risk allowed for the observed impact of vaccination to become apparent. In a Spanish study involving individuals aged 14 years to 23 years, a decline in the prevalence of GWs was noted starting from the year the vaccination cohort reached the ages of 18 years to 19 years [17]. Here, the effect was more pronounced for girls born in 2003 than for those born in 2004, with HR values and statistical significance for the 2004 cohort appearing 1 year later than for the 2003 cohort. This implies that the vaccine’s effectiveness becomes evident after reaching a certain age.

### Strengths and limitations

This study has several strengths. First, its importance lies in its use of representative data encompassing nearly the entire population of HPV vaccine beneficiaries under the NIP. The research utilized claims data from the national health insurance system, which covers approximately 98% of the Korean population. The analysis incorporated healthcare service utilization data, categorized by fee-for-service information, and socioeconomic status, determined by health insurance contributions. In Korea, healthcare providers submit claims and vaccination data electronically, resulting in minimal instances of missing information [23,24]. However, relying solely on the principal diagnosis in claims data can lead to the omission of additional diagnoses [25]. To address this, our study incorporated all recorded diagnostic names. Even though relatively few cases included information on the onset of GWs among adolescents with limited HPV exposure, the results were obtained without a loss of statistical power. This research offers generalizable evidence through a cohort study design that utilizes individual-level data. Moreover, calculating the time-stratified HR is particularly valuable as it reveals outcomes that are influenced by the details of the follow-up period, independent of changes in the variables themselves.

This study also has a few limitations. First, the claims data were not originally collected for research purposes; hence, the ICD-10 codes may not be consistent with the patients’ medical records [26]. To improve the validity of patient definition using disease classification codes for diagnosis, we conducted a review of standard therapies and consulted with clinical experts. Second, additional confounding factors may exist beyond the control variables considered. The unvaccinated group included those who declined vaccination, introducing the potential for fundamental differences in characteristics such as clinical conditions and health behaviors [27]. Additionally, a link may exist between vaccination and sexual activity, or factors affecting sexual activity could be influential. However, it is challenging to formulate specific hypotheses based on previous studies and the available data. Third, interpreting the effects of vaccination was limited by the young age of the study cohort and the brief follow-up period. Although data on vaccinations administered before NIP implementation were not included, this omission is unlikely to meaningfully impact the results, given the clinical guidelines and the vaccination rate among girls at that time [28,29].

Additionally, survival analysis was conducted considering the varying follow-up periods across individuals stemming from differences in vaccination schedules. However, the hazard scale had limitations in causal interpretation compared with risk-based measures. Notably, the analyzed population was conditionally defined for each stratified period to address time-dependent effects, which limited the generalizability of the results.

In conclusion, this study demonstrated that HPV vaccination is associated with a reduction in the risk of GWs among adolescent girls. This reduction became significant as the incidence of GWs increased with participant age. In the short term, the NIP of Korea can be considered effective in providing protection against GWs. However, given that GWs are not typically regarded as a serious disease, it is essential to assess the effectiveness of the NIP in preventing more critical conditions, such as precancerous lesions or cancer. The findings of this study are based solely on an analysis of adolescent girls; therefore, additional research is warranted to include older age groups, specifically those 20 years and older, and to involve men participants.

## Figures and Tables

**Figure f3-epih-46-e2024040:**
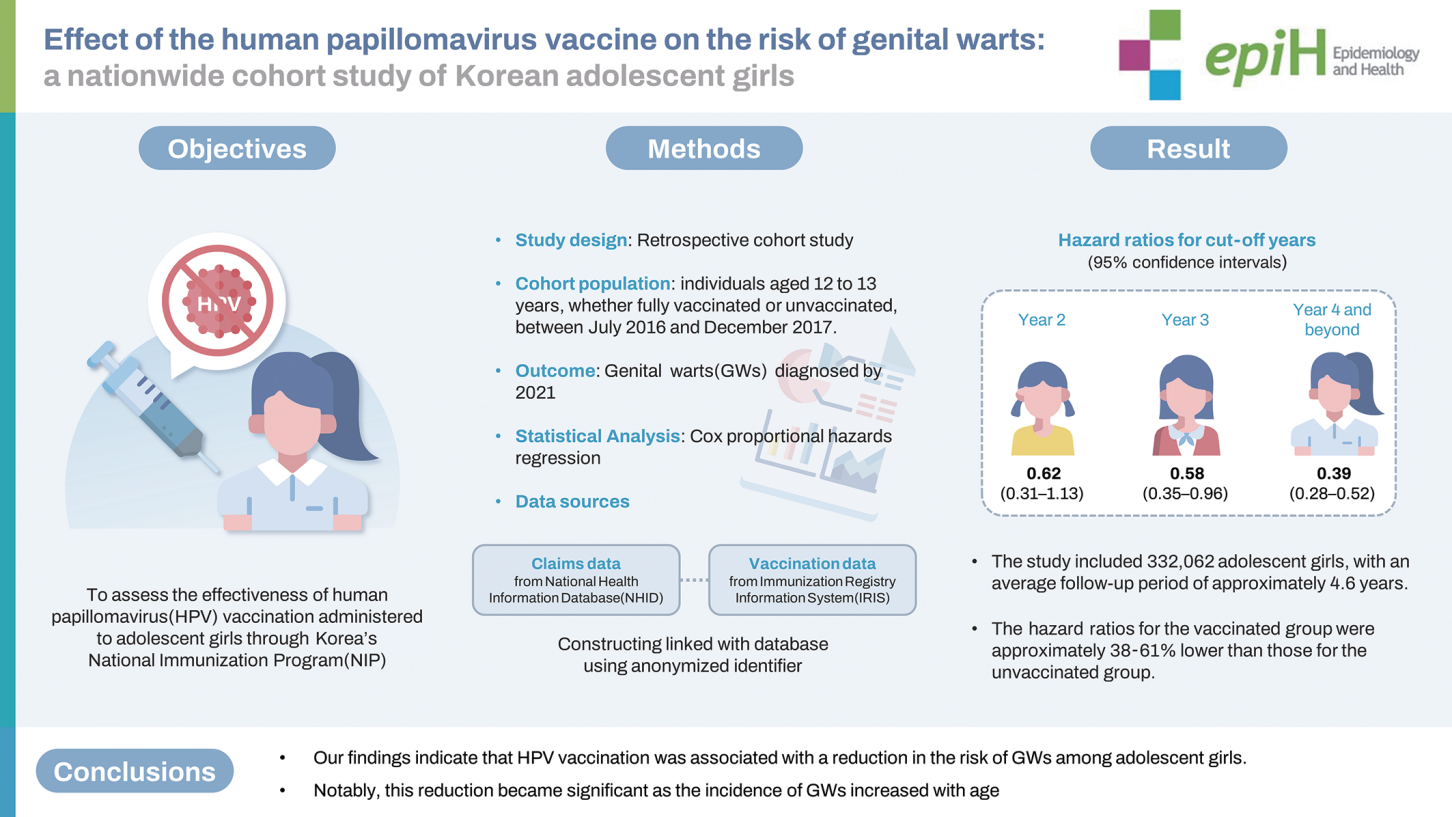


**Table 1.** Baseline characteristics of the study cohort

Characteristics	Before matching			After matching		
	HPV-vaccinated group	HPV-unvaccinated group	p-value	HPV-vaccinated group	HPV-unvaccinated group	p-value
Total no. of individuals	184,359 (50.6)	180,032 (49.4)	-	166,031 (50.0)	166,031 (50.0)	-
Follow-up duration (yr)	4.58±0.30	4.66±0.37	-	4.58±0.30	4.58±0.30	-
Birth year			<0.05			N.S.
2003	86,830 (47.1)	91,881 (51.0)		84,187 (50.7)	84,187 (50.7)	
2004	75,369 (40.9)	62,065 (34.5)		60,795 (36.6)	60,795 (36.6)	
2005	22,160 (12.0)	26,086 (14.5)		21,049 (12.7)	21,049 (12.7)	
Age (at date of first follow-up)	13.30±0.68	13.24±0.78	<0.05	13.32±0.70	13.31±0.70	N.S.
Birth year 2003	13.87±0.38	13.85±0.44		13.86±0.38	13.86±0.38	
Birth year 2004	12.90±0.40	12.84±0.42		12.89±0.40	12.89±0.40	
Birth year 2005	12.38±0.33	12.14±0.60		12.37±0.33	12.33±0.32	
Age (at date of last follow-up)	17.88±0.74	17.83±0.78	<0.05	17.85±0.75	17.86±0.76	N.S.
Birth year 2003	18.52±0.30	18.46±0.31		18.47±0.30	18.47±0.31	
Birth year 2004	17.53±0.30	17.47±0.30		17.48±0.30	17.47±0.30	
Birth year 2005	16.56±0.30	16.46±0.31		16.51±0.30	16.46±0.30	
Type of vaccine			-			-
4-valent	184,093 (99.9)	N/A		165,831 (99.9)	N/A	
9-valent	266 (0.1)	N/A		200 (0.1)	N/A	
Socioeconomic status			<0.05			N.S.
Medical Aid	5,714 (3.1)	6,240 (3.5)		5,266 (3.2)	5,266 (3.2)	
Lowest	41,928 (22.7)	40,012 (22.2)		36,827 (22.2)	36,827 (22.2)	
Low	28,803 (15.6)	28,143 (15.6)		25,754 (15.5)	25,754 (15.5)	
Middle	33,850 (18.4)	32,082 (17.8)		29,966 (18.1)	29,966 (18.1)	
High	40,795 (22.1)	38,344 (21.3)		36,519 (22.0)	36,519 (22.0)	
Highest	33,269 (18.1)	35,211 (19.6)		31,699 (19.1)	31,699 (19.1)	
Regional urbanization level			<0.05			<0.05
Metropolis	113,038 (61.3)	112,221 (62.3)		104,691 (63.1)	107,670 (64.8)	
Urban	58,215 (31.6)	56,777 (31.5)		50,867 (30.6)	49,370 (29.7)	
Rural	13,106 (7.1)	11,034 (6.1)		10,473 (6.3)	8,991 (5.4)	

Values are presented as number (%) or mean±standard deviation. HPV, human papillomavirus; N/A, not applicable; N.S., not significant.

**Table 2.** Hazard ratios (HRs) for genital warts

Subgroup	Follow-up period; Baseline to cut-off year (conditional status)	Vaccination status	n	Person-years	No. of event cases	HR (95% CI)^1^
All	Baseline to Year 1 (entire population)	Y	166,031	166,021	13	1.29 (0.57, 2.94)
		N	166,031	166,014	10	1.00 (reference)
	Baseline to Year 2 (first-year survivors)^2^	Y	165,976	165,971	13	0.62 (0.31, 1.13)
		N	165,976	165,963	21	1.00 (reference)
	Baseline to Year 3 (second-year survivors)^2^	Y	165,895	165,889	23	0.58 (0.35, 0.96)
		N	165,895	165,884	40	1.00 (reference)
	Baseline to Year 4 and beyond (third-year survivors)^2^	Y	165,788	138,063	57	0.39 (0.28, 0.52)
		N	165,788	138,047	148	1.00 (reference)
Birth year 2003	Baseline to Year 1 (entire population)	Y	84,187	84,180	9	1.29 (0.48, 3.46)
		N	84,187	84,179	7	1.00 (reference)
	Baseline to Year 2 (first-year survivors)^2^	Y	84,157	84,153	8	0.44 (0.19, 1.01)
		N	84,157	84,149	18	1.00 (reference)
	Baseline to Year 3 (second-year survivors)^2^	Y	84,101	84,098	12	0.48 (0.24, 0.96)
		N	84,101	84,095	25	1.00 (reference)
	Baseline to Year 4 and beyond (third-year survivors)^2^	Y	84,039	71,052	36	0.38 (0.26, 0.55)
		N	84,039	71,043	96	1.00 (reference)
Birth year 2004	Baseline to Year 1 (entire population)	Y	60,795	60,794	2	N/A^3^
		N	60,795	60,790	2	
	Baseline to Year 2 (first-year survivors)^2^	Y	60,779	60,778	4	N/A^3^
		N	60,779	60,776	2	
	Baseline to Year 3 (second-year survivors)^2^	Y	60,763	60,761	7	0.63 (0.25, 1.63)
		N	60,763	60,760	11	1.00 (reference)
	Baseline to Year 4 and beyond (third-year survivors)^2^	Y	60,734	51,061	20	0.44 (0.26, 0.75)
		N	60,734	51,056	45	1.00 (reference)

CI, confidence interval; Y, yes (vaccinated); N, no (unvaccinated).

1 HR adjusted for birth year, socioeconomic status, and regional urbanization level.

2 Prior to each stratified period, the following were excluded: persons diagnosed with genital warts, persons who had been censored, and their matched counterparts.

3 Results were not estimated due to an insufficient number of events.

## Supplementary materials

Supplementary Material 1. Diagnosis of HPV-related disease within 5 years prior to the start of the follow-up period

Supplementary Material 2. Kaplan-Meier analysis of overall survival without time-stratification
