# Supplementary material for: Effect of the human papillomavirus vaccine on the risk of genital warts: a nationwide cohort study of Korean adolescent girls
Source: Epidemiol Health. 2024 Mar 18;46:e2024040. doi: 10.4178/epih.e2024040 (PMC11369562; doi:10.4178/epih.e2024040)

Supplementary Material 1. Diagnosis of HPV-related disease within 5 years prior to the start of the follow-up period


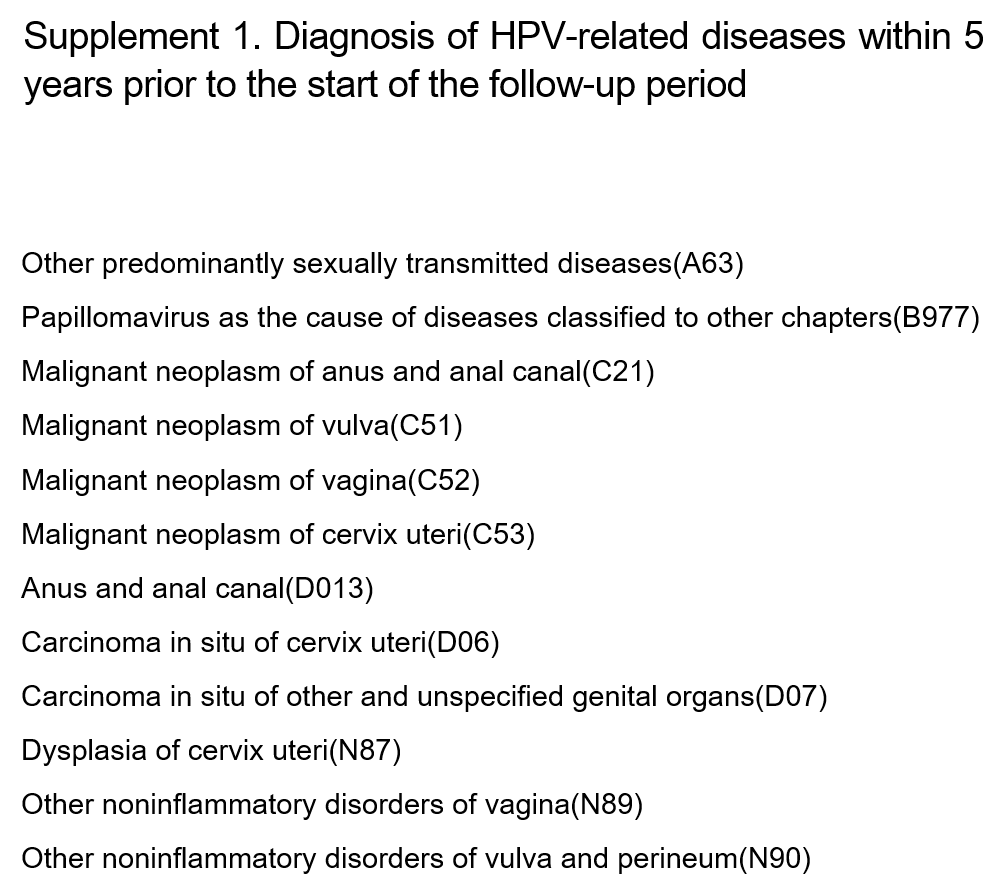

Supplement: Supplementary Material 1. — Diagnosis of HPV-related disease within 5 years prior to the start of the follow-up period [file epih-46-e2024040-Supplementary-1.docx]
