# Supplementary material for: Effect of the human papillomavirus vaccine on the risk of genital warts: a nationwide cohort study of Korean adolescent girls
Source: Epidemiol Health. 2024 Mar 18;46:e2024040. doi: 10.4178/epih.e2024040 (PMC11369562; doi:10.4178/epih.e2024040)

Supplementary Material 2. Kaplan-Meier analysis of overall survival without time-stratification


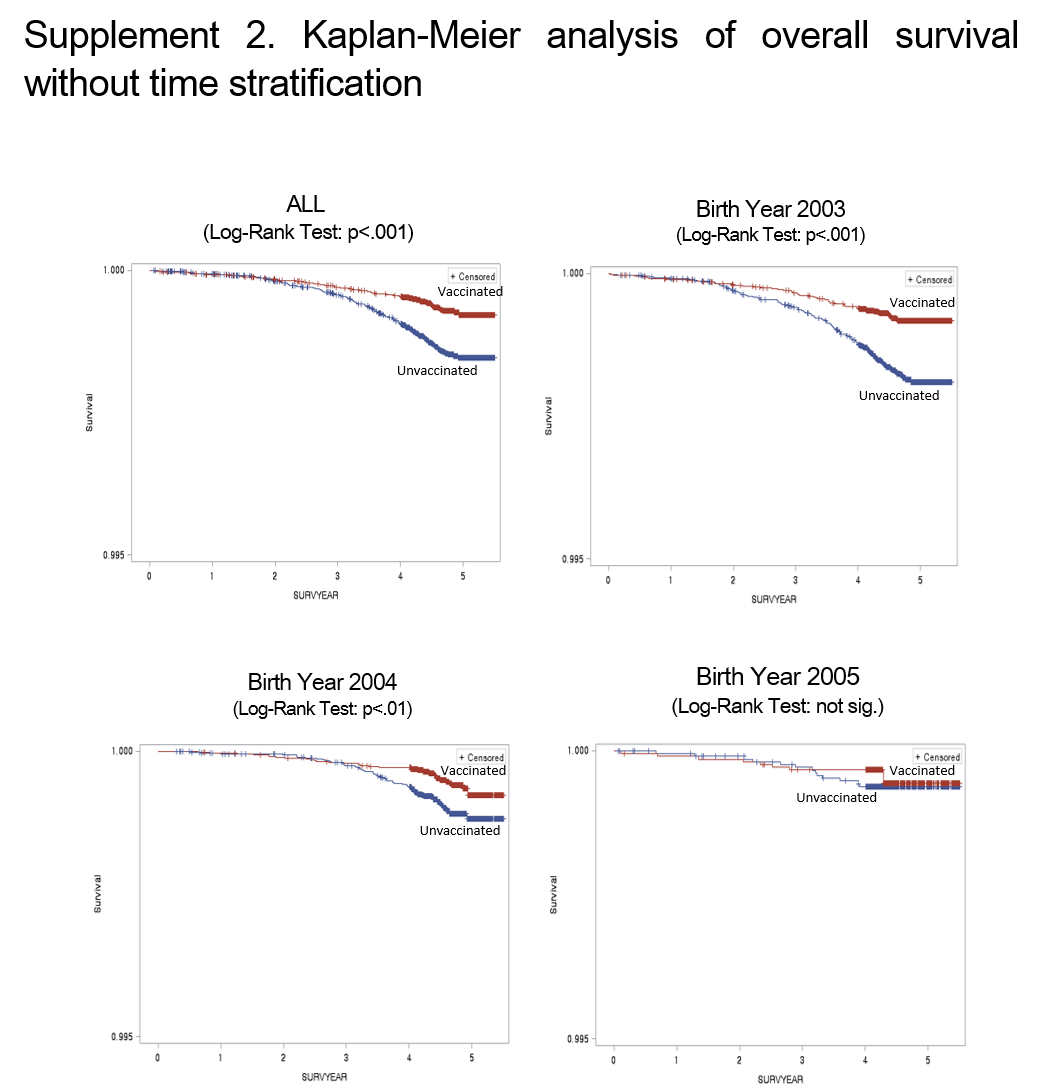

Supplement: Supplementary Material 2. — Kaplan-Meier analysis of overall survival without time-stratification [file epih-46-e2024040-Supplementary-2.docx]
